# Choroidal and Retinal Thicknesses in Healthy Eyes Measured with Ultra-Wide-Field Optical Coherence Tomography

**DOI:** 10.3390/diagnostics14111114

**Published:** 2024-05-28

**Authors:** Krzysztof Kiciński, Maciej Gawęcki

**Affiliations:** 1 Department of Ophthalmology, Pomeranian Hospitals, 84-120 Wejherowo, Poland; krzysztofkg999@icloud.com; 2 Dobry Wzrok Ophthalmological Clinic, 80-822 Gdansk, Poland

**Keywords:** ultra-wide-field optical coherence tomography, choroidal thickness, retinal thickness, axial length

## Abstract

Ultra-wide-field optical coherence tomography (UWF-OCT) has been recently introduced into clinical ophthalmological practice. To date, there are few data on the reference values of the retinal thickness (RT) and choroidal thickness (CT) measured with this technique. This study aimed to analyze the variance in RT and CT in the healthy eyes of white Caucasian patients with UWF-OCT tests performed with the largest available scan size of 23 × 20 mm. The data were analyzed with reference to the patients’ age and gender and the axial length of the eyeball. The results of UWF-OCT scanning enabled us to visualize the shape of the retina and choroid in a large portion of the eyeball. Both anatomical entities became significantly thinner at the periphery. The peripheral CT was greater in the upper and temporal sectors; the RT was higher in the nasal compared to the temporal sectors. Both the choroid and retina showed a reduced thickness with age; however, the CT and RT did not show a statistically significant correlation with the axial length after adjusting for age and gender. Age-related variations in thickness were especially prominent in the choroid. The CT in UWF-OCT testing was significantly greater in females, while the RT was greater in males. UWF-OCT testing provides additional information on the anatomical structure of the retina and choroid compared to standard-field OCT.

## 1. Introduction

Determining normative databases in chorioretinal diseases is important for the precise evaluation of ocular pathologies. This is especially valid for optical coherence tomography (OCT) devices that provide measurements of the retina and the choroid [1,2]. Thus far, the most popular databases refer to the central retinal thickness (RT) measured within the Early Treatment Diabetic Retinopathy Study (ETDRS) grid and the retinal nerve fiber layer (RNFL) thickness assessed in glaucoma [3,4]. The choroidal thickness (CT) database is not yet available for most OCT devices, partly due to its major variance depending on the patient’s age and the axial length of the eyeball [5]. Nevertheless, up-to-date research provides some data on the CT in healthy individuals, usually limited to the central area of the posterior pole [6]. This is because wide-field (WF) OCT devices have only recently been introduced into clinical practice and are not yet commonly used in ophthalmological clinics. As peripheral retinal changes often correlate with central ocular pathologies, determining the reference thickness values for the peripheral retina and choroid would enable the more precise evaluation of ocular disorders in general [7,8,9].

The goal of our study was to analyze the variance in the RT and CT in healthy eyes with WF-OCT testing performed with the largest available scan size of 23 × 20 mm. The data were analyzed with reference to the patients’ age and gender and the axial length of the eyeball.

## 2. Materials and Methods

The study included 75 consecutive white Caucasian patients admitted to the outpatient ophthalmological clinic at the Specialist Hospital in Chojnice, Poland, between May and June 2023, for a routine ophthalmological check-up, including the prescription of spectacles. All patients underwent a routine ophthalmological examination that included best-corrected visual acuity testing, automated refraction, biomicroscopy of the anterior and posterior segments of the eye and intraocular pressure measurement. The eyes without ocular pathologies or previous ocular surgical treatment were initially qualified for wide-field OCT testing. Cases with a refraction error larger than 6.0D in myopia, 4D in hypermetropia and 3D in astigmatism were excluded. Patients burdened with systemic diseases that could influence the results, especially diabetes mellitus, hypertension, hypercholesterolemia, metabolic disorders or pregnancy, were also excluded from the study group. The implementation of these criteria resulted in the exclusion of 80 patients and 25 associated eyes; thus, altogether, 125 eyes were included in the study. The baseline characteristics of the cohort are presented in Table 1.

**Figure 1:**
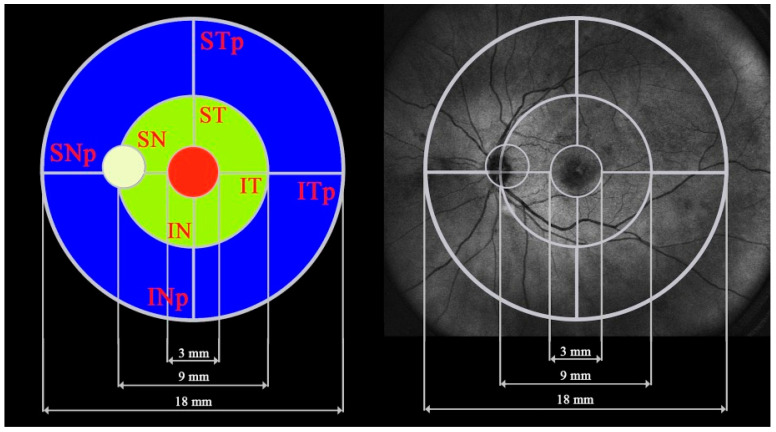
Visualization of the nine fields included in the analysis. SN: superonasal, ST: superotemporal, IT: inferotemporal, IN: inferonasal, SNp: peripheral superonasal, STp: peripheral superotemporal, ITp: peripheral inferotemporal, INp: peripheral inferonasal.

Before the examination, all patients were asked to restrain from consuming coffee or smoking. All scans were obtained at the same time of the day, in the morning, to exclude the impact of the diurnal variability of the RT and CT. Measurements were performed after pupil dilation with a swept-source Xephilio OCT-S1 (Canon Medical Systems Europe B.V.,Amstelveen, Netherlands 2023). The device provides a scanning speed of 100,000 A scans per second, a horizontal resolution of 30 μm, an axial resolution of 8 μm and a scan width range of 3–23 mm. The protocol that was used enabled us to capture a retinal area of 23 × 20 mm (the maximum) with measurements performed in 25 sectors enclosed in a circle of 18 mm in diameter, automatically centered at the fovea. The sector arrangement was provided in the Xephilio device. Each scan was evaluated individually and its centration was corrected if needed. The segmentation of layers was also manually checked and corrected if necessary. The results for neighboring fields were added to represent larger retinal areas. This procedure was performed to enhance the strength of the statistical analysis, as well as for the more detailed presentation of the study outcomes. Altogether, nine fields were analyzed: central, superonasal (SN), superotemporal (ST), inferotemporal (IT), inferonasal (IN), peripheral superonasal (SNp), peripheral superotemporal (STp), peripheral inferotemporal (ITp) and peripheral inferonasal (INp). The central area refers to the central circle of 3 mm in diameter; the SN, ST, IT and IN areas are localized between the central 3 mm circle and the larger 9 mm circle; the SNp, STp, ITp and INp areas are located between the 9 mm circle and the 18 mm circle. The dimensions and shapes of the analyzed retinal areas are shown in Figure 1. The obtained measurement data represent the average thickness values in each of the fields. Only the scans suitable for numeric evaluation were included in the analysis. Additional data obtained from the standard measurement protocol included the axial length of the eyeball in mm.

### Statistical Procedures

Categorical traits were described through integer numbers and percentages. Numerical traits were depicted by using their mean, median, standard deviation and lower-to-upper quartile values. The normality of the distribution was assessed using the Shapiro–Wilk W-test. Levene’s test was used to assess the homogeneity of variances. A multifactor analysis of variance (ANOVA) was performed to test the significance of differences in normally distributed numerical traits between the study groups. When dealing with non-normally distributed quantitative variables, generalized linear models were used. The Pearson product moment correlation coefficient *r* was computed when assessing the relationships between selected numerical traits. All procedures were performed by using Statistica™ release 13 (TIBCO Software Inc., Palo Alto, CA, USA).

## 3. Results

The analysis of the RT and CT across the sectors revealed significant variance between all sectors at *p* < 0.0001 (Table 2 and Table 3). Significant differences were also found in the pairwise comparisons in each pair of measured retinal areas and all but two comparisons in the choroidal areas (Table 4 and Table 5). As expected, the retina and choroid are thickest in the central region. As we move to the periphery, the retinal and choroidal thicknesses are significantly lower (Table 2). Temporal sectors have higher CT values compared to nasal sectors; superior quadrants have greater thickness values than inferior quadrants. Conversely, the RT is higher in nasal sectors compared to temporal ones. A greater CT in the central 3 mm area is correlated positively with the retinal thickness in this region (r = 0.21; *p* = 0.0116).

In general, male patients had thicker retinas than female patients. This difference was significant for the central and paracentral portions of the retina but not the far peripheral, where the thickness values were similar in both genders. Conversely, females had a greater choroidal thickness than males. Although the difference was not significant in the central area of 3 mm diameter, it was very apparent in all other sectors, including the far periphery (Table 6).

**Figure 2:**
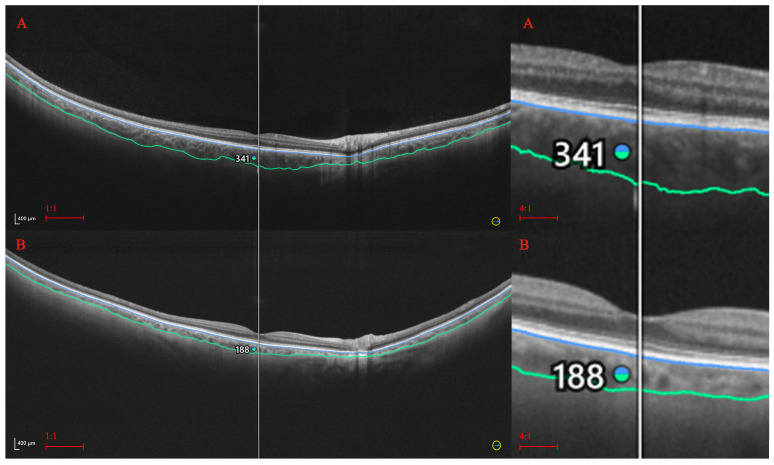
Choroidal thickness in young (**A**) and older (**B**) individuals. A significant difference in the central CT is visible on the scans. The blue line corresponds to Bruch’s membrane and green to choroid-sclera interface.

**Figure 3:**
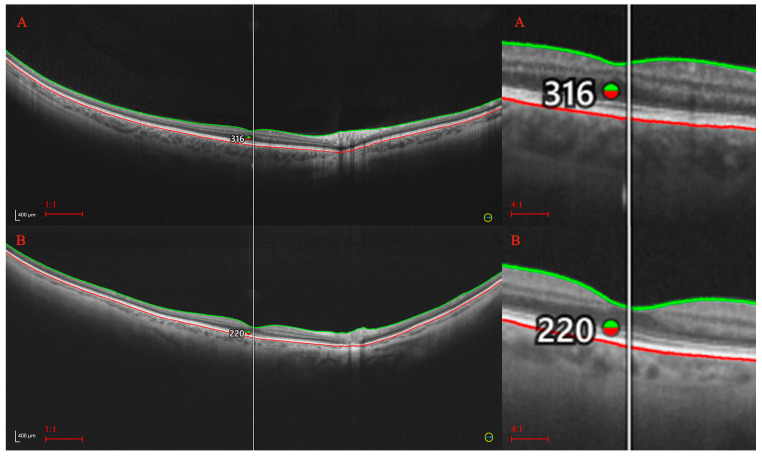
Retinal thickness in young (**A**) and older (**B**) individuals. A significant difference in the central RT is marked on the UWF-OCT scans. Green line refers to internal limiting membrane and red to the RPE base.

The analysis of the retinal and choroidal thicknesses by age provided straightforward results for the central and paracentral sectors. The sectoral retinal and choroidal thicknesses in these areas diminish with age (Table 7). Such variation is especially prominent in the choroid. The difference between the central CT in patients younger than 40 and those in their 80s is approximately 100 μm, which is almost 30% of the baseline value. For the retina, such a correlation is also significant but less apparent numerically. It seems that significant retinal thinning (total and sectoral) occurs late, i.e., in the eighth decade of life. A clear difference in the RT was observed between patients over 70 and those under 40, although it did not exceed 14 μm (4% of the baseline value) in any of the measured sectors. Moreover, the retinal areas located at the far periphery did not show strong thickness variations with age. Examples of the differences in CT and RT between young and older individuals are presented in Figure 2 and Figure 3.

The mean axial length value was 24.79 ± 1.60 mm and generally did not correlate with the RT or CT after adjusting for age and gender (Table 8).

## 4. Discussion

Most of the studies that analyzed the retinal and choroidal thickness variation used traditional equipment with a standard field of view. Thus, most of the data refer to the area of the ETDRS grid with a maximum diameter of 6 mm or a wider field of 12 mm width but rarely with the use of wide-field OCT scanning [10]. In our study, performed with wide-field equipment and 23 × 20 mm scans, we analyzed the CT and RT values outside the central part of the posterior globe and provided additional data for these considerations. To our knowledge, this is the widest field of view available at present in OCT devices with the widest numerically analyzed area of 18 mm in diameter and, as such, it enables us to provide a more extensive picture of the architecture of the globe. Only a few other studies with the use of UWF-OCT, without the need to create a mosaic of images, have been performed so far [11,12]. In our study, the choroid had the shape of a convex–concave lens, with the peripheral thickness higher at the temporal side compared to the nasal side and the upper side compared to the inferior side. The retina had an approximately double convex shape with a thicker part located around the optic nerve. A similar picture of the retinal and choroidal architectures analyzed by UWF testing was presented by Hirano et [11]. In other studies, such a difference between the architectures of these anatomical entities was also identified, but in standard-field OCT examinations. For example, Wang et al. analyzed variations in the central 6 mm circle (ETDRS grid) [13]. Rasheed et al. used a mosaic of OCT scans to obtain a wider field of view [14]. The authors noted a higher mean CT in vertical scans compared to horizontal ones and a smaller CT in all peripheral sectors, especially the inferior ones, which is consistent with our data.

Former studies on the variation in the CT in healthy individuals with the use of standard-field OCT showed a similar percentage of symmetrical versus asymmetrical patterns and quite a high percentage of thick choroids in younger patients (subfoveal CT > 395 μm in 30% of cases younger than 55 years) [15]. This finding is confirmed by the results of our study: the third quartile value for the mean CT in the foveal area (3 mm in diameter) in patients younger than 40 was 395.50 μm, meaning that 25% of this age group had a higher CT.

One of the few WF-OCT studies, conducted by Kim et al., found a symmetrical CT in both eyes in healthy individuals [12]. Nevertheless, differences in the CT values between the eyes were greater at the periphery, indicating greater anatomical variations in the peripheral areas. In our study, the anatomical variation in thickness was similar for the central and peripheral areas.

### 4.1. Gender-Related Variations in RT and CT

In our study, men generally had a greater retinal thickness compared to women, whereas women had a significantly larger choroidal thickness compared to men. The CT in the central 3 mm part of the macula was similar in males and females; however, the peripheral sectors had significantly higher mean CT values in females. The use of UWF-OCT provides the possibility to show this difference, contrary to standard-field imaging. This topic has also been analyzed in other studies, although usually without WF-OCT. Wang et al. reported higher mean RT and CT values in men in an analysis that included only the central 6 mm circle corresponding to the ETDRS grid. On the other hand, a study by Zhang et al. with 12 × 9 mm scans did not report gender-related variations in RT and CT. A similar outcome was presented in Rasheed’s study on healthy eyes, which involved a mosaic of OCT scans using Heidelberg Spectralis. It is plausible that, without including measurements of the choroidal periphery, such gender-related differences in CT and RT were not recorded. Our study suggests a greater volume of the whole choroid in females and a greater retinal volume in males.

### 4.2. Age-Related Variations in RT and CT

An important consideration is the relation of the retinal and choroidal thicknesses to the patient’s age, especially considering that normal choroidal thickness values strongly impact analyses in the context of pachychoroid spectrum diseases. In our study, both the CT and RT were generally strongly correlated with the patient’s age; the exceptions were the far peripheral retinal areas, which did not show such a strong variation. The correlation was particularly apparent for the choroid, which was significantly reduced in thickness over time in all sectors. Thus, CT evaluation in the pachychoroid spectrum must take into account the choroidal thinning occurring with older age. This also concerns the peripheral choroid, whose thickness is also clearly dependent on the patient’s age. This finding may not be surprising but is novel in the context of the available research, which has employed only standard-field OCT imaging.

Variations in the choroidal and retinal thicknesses with age were analyzed in a few studies. Similar results to ours were reported by Hirano et al. for UWF-OCT [11]. In a study of the ETDRS grid area, Pongsachareonnont et al. also found a significant reduction in retinal and choroidal thickness with age; only the nerve fiber layer thickness was age-independent. Similar findings were reported for the choroid by Xie et al. and Cortes et al. in studies with standard-field OCT [5,16]. The authors reported significant CT loss over the age of 50. Wang et al. also reported the loss of total RT and CT in older patients. Zhang noted lower CT values in older people using SS-OCT 12 × 9 mm scans [17]. A thinner peripapillary choroid in older individuals was also reported by Yang et al. [18]. The authors calculated a loss of 9 μm in the total CT per decade. On the other hand, Rasheed et al. did not find CT variations with age. Such a lack of age dependence for the RT and CT is seldom reported.

### 4.3. Axial Length and the RT and CT

Our study did not find a significant influence of the axial length on the measurement of the RT and CT after adjusting for age and gender. This result refers also to the far periphery of the retina and choroid. The data on such correlations vary among studies. Mansoori et al., Yao et al. and Abbey et al. reported a thinner central choroid and retina in longer eyeballs [19,20,21]. All of these studies used standard-field OCT scans. On the other hand, in a wide-field study, Hirano et al. found a weak negative correlation for selected sectors of the choroid. Nadeem did not find such a correlation in a large group of children [22]. Our analysis differed from most previous studies as it applied both multifactor statistical modeling (age and gender adjustment) and wide-field OCT testing. As such, and given the lack of large studies employing UWF-OCT, the present study should be treated as a stand-alone research work and should not be simply compared with data from other papers.

### 4.4. CT and RT and Ocular Diseases

The retinal and choroidal thickness are strongly associated with the occurrence of ocular diseases. This refers to both straightforward situations, such as typical retinal edema, but also variations in retinal thickness in general, which can precede disease onset, represent a risk factor for its development or determine its course. Such variations have been reported in such clinical entities as diabetic retinopathy or retinal vein occlusion [23,24,25]. The analysis of the choroidal thickness led to the identification of a spectrum of pachychoroid disorders, in which an increased CT and congestion of the choriocapillaris underly the mechanism of penetration of serous fluid under a neurosensory retina [26,27]. The visualization and measurement of the CT can also supply information on the state of the ocular circulation or its deficit, which has a correlation with the onset of degenerative diseases such as retinal degeneration and dystrophy [28,29,30]. CT measurements have potential in the monitoring of disease activity in the follow-up of inflammatory choroidal disorders or immunological choroidopathies, as well as choroidal tumors or granulomas [31,32,33].

For all such analyses, a normative database is necessary to serve as a reference. Despite the large variations in the RT and CT according to age or gender, it is important to outline the possible ranges of these thicknesses that would constitute a benchmark. Wide-field OCT systems have only recently been introduced to clinical practice, so information on the far peripheral retinal and choroidal thickness is still a subject for analysis. The normative database for the peripheral RT and CT can stand as a basis for further research on the involvement of these regions in ocular pathologies. Future research may also explore variations in the peripheral RT and CT in systemic diseases, such as diabetes or hypertension, and their reference to healthy populations.

At this point, it is worth noting that new developments in spectral optical coherence tomography technologies are under research—for example, hyperspectral systems, which measure thousands of spectra [34]. They combine the spatial pixel and extended spectral information to obtain three-dimensional data comprising position-, wavelength- and time-related details. The high resolution and dynamics of the procedure provide more knowledge of the tissue properties and composition, as well as changes in its physiological parameters. Hopefully, the development of these devices will enable the live monitoring of disease evolution.

### 4.5. Limitations of the Study

The study provides RT and CT values for the white Caucasian population only. It cannot be excluded that differences in these measurements exist among different races, as has been suggested by other research [35]. This should be studied in further clinical trials.

## 5. Conclusions

Ultra-wide-field optical coherence tomography (UWF-OCT) scanning enables one to visualize the shape of the retina and choroid in a large portion of the eyeball. In general, the UWF picture of the architecture of the globe confirms the findings of standard-field studies. Both anatomical entities become significantly thinner at the periphery and this trend is preserved on UWF-OCT scans at the far peripheral areas. The peripheral choroid thickness (CT) is greater in the upper and temporal sectors. Both the choroid and retina show reduced thickness with age; however, the CT and retinal thickness (RT) do not show a statistically significant correlation with the axial length after adjusting for age and gender. Age-related variations refer to both the central and peripheral regions and are especially prominent for the choroid. The CT on UWF-OCT testing is significantly greater in females and the RT is greater in males.

## Figures and Tables

**Table 1:** Baseline characteristics of the control group (discrete variables, *n* = 75 individuals).

Analyzed Trait	No. (Percentage in %)
No. of participants	75 (50.68)
No. of eyes	125 (43.60)
Gender:	
- Female	48 (64.00)
- Male	27 (36.00)
Age group (years):	
- Up to 40	24 (19.20)
- 41–50	12 (9.60)
- 51–60	33 (26.40)
- 61–70	20 (16.00)
- Over 70	36 (28.80)

**Table 2:** Descriptive statistics for the parafoveal retinal thickness (µm) in the control group (*n* = 125 eyes).

Retinal Thickness (µm)	Statistical Parameter				*p*-Value *
	M	SD	Me	Q_1_–Q_3_	
Central	335.08	20.69	335.00	324.00–348.00	<0.0001
ST	271.90	14.10	273.75	262.00–282.00	
SN	309.65	16.69	311.50	299.00–320.25	
IN	300.99	17.91	302.50	289.25–311.50	
IT	267.42	16.51	268.75	259.00–278.00	
STp	212.32	10.60	212.00	205.00–220.00	
SNp	242.94	16.29	243.50	232.00–254.50	
INp	220.38	15.42	219.50	210.00–230.00	
ITp	205.52	11.69	206.00	199.00–214.00	

* Controlled for the study subjects’ gender. SN: superonasal, ST: superotemporal, IT: inferotemporal, IN: inferonasal, SNp: peripheral superonasal, STp: peripheral superotemporal, ITp: peripheral inferotemporal, INp: peripheral inferonasal, M: mean, Me: median, SD: standard deviation, Q: quartile.

**Table 3:** Descriptive statistics for the parafoveal choroidal thickness (µm) in the control group (*n* = 125 eyes).

Choroidal Thickness (µm)	Statistical Parameter				*p*-Value *
	M	SD	Me	Q_1_–Q_3_	
Central	293.90	87.10	300.00	223.00–357.00	<0.0001
ST	283.94	77.20	285.00	223.25–348.00	
SN	244.60	75.52	245.25	184.00–307.75	
IN	218.72	75.10	204.25	162.75–289.25	
IT	256.65	79.29	246.00	192.00–312.75	
STp	225.16	54.80	219.00	183.50–261.50	
SNp	192.38	57.77	189.00	151.50–237.50	
INp	139.98	39.78	129.50	107.50–163.00	
ITp	188.75	48.50	179.00	150.00–226.00	

* Controlled for the study subjects’ age and gender. SN: superonasal, ST: superotemporal, IT: inferotemporal, IN: inferonasal, SNp: peripheral superonasal, STp: peripheral superotemporal, ITp: peripheral inferotemporal, INp: peripheral inferonasal, M: mean, Me: median, SD: standard deviation, Q: quartile.

**Table 4:** Comparisons between retinal thickness measurements.

RT	Central	ST	SN	IN	IT	STp	SNp	INp
ST	<0.0001							
SN	<0.0001	<0.0001						
IN	<0.0001	<0.0001	<0.0001					
IT	<0.0001	<0.0001	<0.0001	<0.0001				
STp	<0.0001	<0.0001	<0.0001	<0.0001	<0.0001			
SNp	<0.0001	<0.0001	<0.0001	<0.0001	<0.0001	<0.0001		
INp	<0.0001	<0.0001	<0.0001	<0.0001	<0.0001	<0.0001	<0.0001	
ITp	<0.0001	<0.0001	<0.0001	<0.0001	<0.0001	<0.0001	<0.0001	<0.0001

RT: retinal thickness, SN: superonasal, ST: superotemporal, IT: inferotemporal, IN: inferonasal, SNp: peripheral superonasal, STp: peripheral superotemporal, ITp: peripheral inferotemporal, INp: peripheral inferonasal.

**Table 5:** Comparisons between choroidal thickness measurements.

CT	Central	ST	SN	IN	IT	STp	SNp	INp
ST	=0.0148							
SN	<0.0001	<0.0001						
IN	<0.0001	<0.0001	<0.0001					
IT	<0.0001	<0.0001	<0.0001	<0.0001				
STp	<0.0001	<0.0001	<0.0001	=0.1744	<0.0001			
SNp	<0.0001	<0.0001	<0.0001	<0.0001	<0.0001	<0.0001		
INp	<0.0001	<0.0001	<0.0001	<0.0001	<0.0001	<0.0001	<0.0001	
ITp	<0.0001	<0.0001	<0.0001	<0.0001	<0.0001	<0.0001	=0.2615	<0.0001

CT: choroidal thickness, SN: superonasal, ST: superotemporal, IT: inferotemporal, IN: inferonasal, SNp: peripheral superonasal, STp: peripheral superotemporal, ITp: peripheral inferotemporal, INp: peripheral inferonasal.

**Table 6:** Retinal and choroidal thicknesses in the control group by gender (numerical variables, *n* = 125 eyes).

Analyzed Trait	Gender	Statistical Parameter *				*p*-Value **
		M	SD	Me	Q_1_–Q_3_	
Central RT (µm)	Male	340.43	23.86	342.50	332.00–360.00	=0.0191
	Female	332.17	18.26	334.00	323.00–345.00	
ST	Male	274.93	14.61	276.62	262.12–283.88	=0.0007
	Female	270.26	13.62	272.25	262.00–278.50	
SN	Male	312.35	17.05	313.87	301.87–324.63	=0.0013
	Female	308.18	16.41	311.00	297.75–319.75	
IN	Male	306.52	16.79	305.62	296.62–317.75	=0.0148
	Female	297.98	17.87	299.00	287.75–310.25	
IT	Male	271.19	16.67	273.25	260.37–280.38	=0.0145
	Female	265.44	16.17	267.00	258.75–275.00	
STp	Male	216.30	10.23	217.00	208.00–223.75	=0.1218
	Female	210.17	10.23	210.00	203.50–215.50	
SNp	Male	249.59	15.53	250.25	238.00–259.75	=0.0241
	Female	239.32	15.63	238.50	228.00–247.50	
INp	Male	227.05	12.61	227.25	217.75–232.25	=0.5537
	Female	216.75	15.66	216.00	205.50–227.50	
ITp	Male	209.75	10.18	211.50	201.25–217.00	=0.3419
	Female	203.23	11.87	203.00	197.00–212.00	
Central CT (µm)	Male	284.70	79.44	289.00	218.00–343.50	=0.5435
	Female	298.89	91.08	300.00	227.00–359.00	
ST	Male	263.13	68.92	265.87	208.50–318.38	<0.0001
	Female	295.25	79.48	301.50	237.25–359.50	
SN	Male	239.19	73.25	232.75	175.12–293.38	=0.0009
	Female	247.53	77.02	247.25	197.50–309.75	
IN	Male	218.36	66.08	213.87	161.12–281.13	=0.0005
	Female	218.91	79.98	200.50	162.75–291.25	
IT	Male	239.76	65.72	240.75	186.12–281.88	<0.0001
	Female	265.82	84.74	251.75	195.25–336.50	
STp	Male	211.97	44.98	209.50	180.75–248.25	<0.0001
	Female	232.33	58.48	232.00	184.50–269.50	
SNp	Male	179.68	44.73	171.50	145.50–202.00	=0.0106
	Female	199.28	62.94	195.00	151.50–243.00	
INp	Male	133.90	27.82	127.75	112.50–151.00	=0.0131
	Female	143.28	44.79	130.00	107.00–175.50	
ITp	Male	175.33	41.51	159.75	144.50–217.75	<0.0001
	Female	196.04	50.67	185.00	155.50–230.50	

* Statistical parameters used: M: mean, SD: standard deviation, Me: median, Q: quartiles. ** Controlled for the study subjects’ age. RT: retinal thickness, CT: choroidal thickness, SN: superonasal, ST: superotemporal, IT: inferotemporal, IN: inferonasal, SNp: peripheral superonasal, STp: peripheral superotemporal, ITp: peripheral inferotemporal, INp: peripheral inferonasal.

**Table 7:** Retinal and choroidal thicknesses in the control group by age (numerical variables, *n* = 125 eyes).

Analyzed Trait	Age Group (Years)	Statistical Parameter *				*p*-Value **
		M	SD	Me	Q_1_–Q_3_	
Central RT (µm)	Up to 40	336.55	15.16	340.50	324.25–348.58	=0.0388
	41–50	337.58	12.00	342.50	326.42–247.58	
	51–60	344.00	17.88	344.50	336.08–353.58	
	61–70	338.30	25.51	334.00	328.00–352.67	
	Over 70	326.67	21.97	330.50	311.42–338.17	
ST	Up to 40	276.91	18.78	279.62	276.46–283.79	=0.0017
	41–50	275.44	10.82	277.12	268.60–284.44	
	51–60	278.27	6.82	277.75	272.35–283.17	
	61–70	271.39	13.18	267.75	260.83–280.58	
	Over 70	265.12	13.34	263.12	256.85–272.42	
SN	Up to 40	315.90	19.36	320.00	303.48–328.65	=0.0041
	41–50	314.85	14.70	315.50	308.87–321.88	
	51–60	314.96	9.75	315.12	306.29–319.75	
	61–70	307.53	15.16	308.25	298.25–317.08	
	Over 70	302.87	17.29	301.00	291.56–318.00	
IN	Up to 40	304.12	23.71	312.12	288.60–321.83	=0.0072
	41–50	306.54	13.08	309.62	303.92–315.44	
	51–60	307.06	11.99	304.12	297.31–315.21	
	61–70	301.45	17.19	299.50	291.67–310.33	
	Over 70	293.10	17.31	294.00	282.25–307.42	
IT	Up to 40	270.44	22.21	274.62	269.67–282.31	=0.0129
	41–50	271.71	11.37	272.87	262.35–279.46	
	51–60	272.56	7.98	270.37	267.00–278.85	
	61–70	269.33	15.00	267.00	258.67–279.25	
	Over 70	259.56	17.13	260.25	249.31–268.33	
STp	Up to 40	213.00	11.78	212.00	209.92–220.25	=0.1967
	41–50	213.73	9.33	210.75	206.42–222.17	
	51–60	214.00	7.48	214.50	206.92–220.79	
	61–70	214.15	11.27	214.50	205.00–220.67	
	Over 70	208.78	10.64	208.25	200.00–216.38	
SNp	Up to 40	244.43	18.35	245.00	236.63–250.96	=0.0141
	41–50	243.81	14.66	244.50	233.13–257.83	
	51–60	247.29	12.61	250.50	236.13–255.67	
	61–70	248.82	18.22	251.00	232.50–259.67	
	Over 70	234.68	12.33	233.50	228.00–241.79	
INp	Up to 40	220.88	18.49	224.00	210.45–231.29	=0.3508
	41–50	218.46	11.94	218.75	210.00–228.29	
	51–60	220.21	9.37	221.00	212.46–228.75	
	61–70	226.53	17.96	227.50	212.17–238.67	
	Over 70	215.79	13.45	215.75	206.42–225.75	
ITp	Up to 40	206.23	16.14	206.50	200.71–216.67	=0.3770
	41–50	206.75	9.39	204.75	200.21–212.29	
	51–60	205.67	6.50	205.75	199.75–210.38	
	61–70	208.11	11.17	209.50	199.33–217.00	
	Over 70	201.90	11.68	202.25	194.71–213.29	
Central CT (µm)	Up to 40	345.20	84.63	348.50	309.17–395.50	<0.0001
	41–50	329.00	68.41	321.50	289.17–373.25	
	51–60	288.50	111.02	259.00	196.75–394.42	
	61–70	286.33	66.65	281.00	260.00–338.33	
	Over 70	250.72	87.46	229.50	181.75–325.83	
ST	Up to 40	339.04	70.66	345.37	297.31–380.81	=0.0002
	41–50	311.45	73.91	306.50	254.85–388.38	
	51–60	278.58	80.97	262.75	196.27–359.00	
	61–70	269.54	53.54	266.25	235.25–314.33	
	Over 70	249.98	80.54	238.37	190.44–310.13	
SN	Up to 40	274.55	74.53	293.37	226.90–340.08	=0.0809
	41–50	260.87	63.83	239.12	212.60–321.88	
	51–60	251.52	84.97	246.37	175.87–329.33	
	61–70	241.94	65.57	247.50	206.00–274.67	
	Over 70	217.24	82.63	204.62	144.60–286.25	
IN	Up to 40	252.46	63.59	270.00	218.17–299.73	=0.0217
	41–50	239.48	69.80	211.75	174.79–309.83	
	51–60	223.12	89.06	194.12	146.98–284.35	
	61–70	212.36	92.93	204.50	183.67–263.50	
	Over 70	190.48	81.63	160.75	128.77–227.73	
IT	Up to 40	299.30	72.50	292.62	257.08–342.94	=0.0021
	41–50	289.04	77.39	272.75	222.00–355.63	
	51–60	258.87	107.23	218.50	182.62–653.60	
	61–70	242.96	62.02	240.25	201.67–274.67	
	Over 70	223.15	72.54	193.87	155.35–257.23	
STp	Up to 40	274.23	40.18	259.25	240.08–313.71	<0.0001
	41–50	240.98	54.80	237.75	199.42–271.88	
	51–60	224.79	46.28	234.75	180.12–267.21	
	61–70	209.86	36.25	210.50	196.00–222.50	
	Over 70	201.50	59.76	190.75	154.24–242.13	
SNp	Up to 40	217.75	53.90	234.50	176.58–256.21	=0.2324
	41–50	200.06	52.46	186.00	159.00–256.42	
	51–60	199.96	70.33	162.00	141.37–243.67	
	61–70	179.82	38.77	174.50	157.83–198.17	
	Over 70	183.17	69.71	168.00	129.50–211.29	
INp	Up to 40	161.10	36.41	161.25	134.79–191.08	=0.1168
	41–50	142.52	38.28	129.00	109.67–165.04	
	51–60	141.88	38.64	128.00	109.5–172.83	
	61–70	131.64	24.76	130.00	116.50–148.00	
	Over 70	133.57	50.27	111.00	98.71–150.25	
ITp	Up to 40	219.10	31.22	224.00	196.46–237.92	=0.0029
	41–50	202.63	49.94	182.25	163.42–248.67	
	51–60	195.83	62.17	167.00	149.96–242.67	
	61–70	177.62	39.24	170.50	151.67–198.83	
	Over 70	170.40	49.09	152.50	133.67–194.97	

* Statistical parameters used: M: mean, SD: standard deviation, Me: median, Q: quartiles. ** Controlled for the study subjects’ age and gender. RT: retinal thickness, CT: choroidal thickness, SN: superonasal, ST: superotemporal, IT: inferotemporal, IN: inferonasal, SNp: peripheral superonasal, STp: peripheral superotemporal, ITp: peripheral inferotemporal, INp: peripheral inferonasal.

**Table 8:** Pearson product moment correlation coefficients and corresponding *p*-values for the axial length versus the retinal thickness and choroidal thickness in the control group.

Thickness (µm)	Axial Length (mm)	
	*r*	*p* *
Central RT	–0.09	0.3496
ST	–0.14	0.1523
SN	–0.15	0.1042
IN	–0.19	0.0491
IT	–0.14	0.1408
STp	–0.05	0.6152
SNp	–0.13	0.1729
INp	0.05	0.5798
ITp	–0.12	0.2068
Central CT	–0.06	0.5540
ST	–0.08	0.4502
SN	–0.01	0.9337
IN	–0.03	0.7306
IT	–0.04	0.6514
STp	0.03	0.7675
SNp	–0.03	0.7302
INp	–0.004	0.9673
ITp	0.06	0.5115

* Both the *r* coefficients and *p*-values were adjusted for the patients’ age and gender. RT: retinal thickness, CT: choroidal thickness, SN: superonasal, ST: superotemporal, IT: inferotemporal, IN: inferonasal, SNp: peripheral superonasal, STp: peripheral superotemporal, ITp: peripheral inferotemporal, INp: peripheral inferonasal.

## Data Availability

The original contributions presented in the study are included in the article, further inquiries can be directed to the corresponding author.
